# A Case of Microscopic Polyangiitis Presenting as Cranial Giant Cell Arteritis

**DOI:** 10.31138/mjr.31.4.412

**Published:** 2020-12-28

**Authors:** Gerasimos Evangelatos, George E. Fragoulis, Alexios Iliopoulos

**Affiliations:** 1 Rheumatology Unit, First Department of Propaedeutic Internal Medicine, School of Medicine, National and Kapodistrian University of Athens, Athens, Greece; 2 Rheumatology Department, 417 Army Share Fund Hospital (NIMTS), Athens, Greece

**Keywords:** Ultrasound, microscopic polyangiitis, giant cell arteritis, vasculitis, temporal artery

## Abstract

We present a case of a 63-year old man with microscopic polyangiitis (MPA) in which the initial clinical presentation resembled the cranial form of giant cell arteritis (GCA) (headache, jaw claudication, low grade fever and raised inflammation markers). Ultrasound of both superficial common temporal arteries revealed signs indicative of vessel wall inflammation. Based on clinical picture and compatible imaging findings, treatment with corticosteroids for GCA was started. After initial improvement and steroid tapering, lung infiltrations, mononeuritis of the right peroneal nerve and cutaneous necrosis appeared and p-Antineutrophil cytoplasmic antibodies (ANCA) turned out to be positive. Three intravenous cyclophosphamide pulses for MPA led in disease remission and maintenance treatment with azathioprine followed. Two years later, the patient has no symptoms and laboratory parameters are normal. This case highlights that MPA can affect temporal arteries and can masquerade as cranial GCA.

## INTRODUCTION

Giant cell arteritis (GCA) and microscopic polyangiitis (MPA) are two distinct forms of vasculitis. GCA is a large-vessel vasculitis that can present with two forms that sometimes overlap: the cranial, and the large-vessel GCA.^1^ Cranial GCA is the classical subtype and symptoms include new-onset headache, jaw claudication, tongue pain, scalp tenderness and visual problems. Polymyalgia rheumatica (PMR) can co-exist with both forms of GCA.^1^ In most cases, increased erythrocyte segmentation rate (ESR) and/or C-reactive protein (CRP) are found in laboratory exams. In contrast, MPA is a systemic antineutrophil cytoplasmic antibodies (ANCA)-associated vasculitis (AAV) of small- and medium-sized arteries. MPA can affect many organs and systems, such as the lungs, kidneys, skin, peripheral nervous system and others. However, vasculitis of MPA might be not limited to the aforementioned sites. Herein, we present a case of a middle-aged man with symptoms resembling cranial GCA. Initially he was treated with steroids for GCA, but upon development of other organs/systems involvement, MPA was diagnosed.

## CASE PRESENTATION

**Figure 1.**
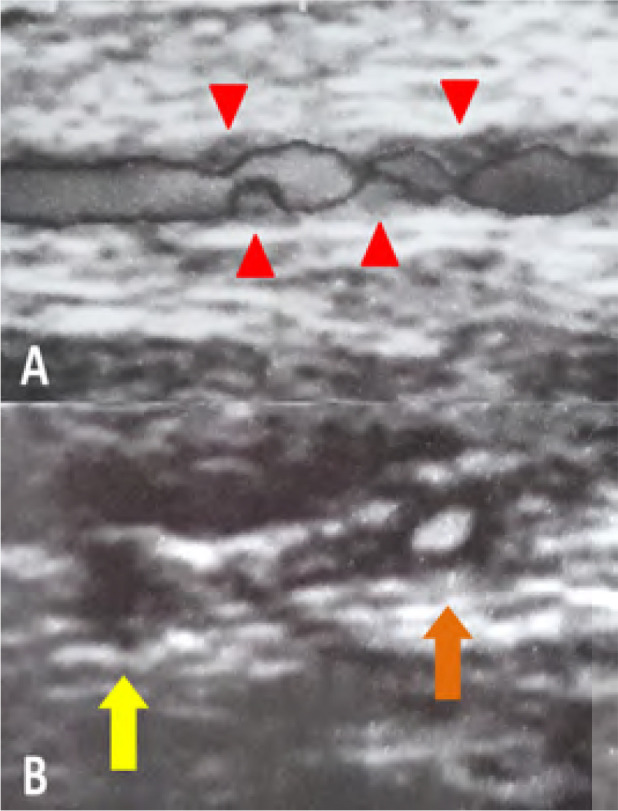
Duplex ultrasonography of superficial common temporal arteries and their main branches in longitudinal (A) and transverse (B) scan. Hypoechoic areas in the vessel wall are shown in the right superficial common artery (A: red arrowheads). A “halo sign” is seen in the parietal branch of the left temporal artery (B: brown arrow), while no blood flow can be detected in the frontal branch (B, yellow arrow).

A 63-year old man presented in our rheumatology clinic complaining about temporal bilateral headache of recent onset, jaw claudication and “PMR-like” symmetrical pain in shoulders and hips. These symptoms were accompanied by malaise and low-grade fever (<38°C). His past medical history and clinical examination was unremarkable. The only abnormal laboratory findings were elevated inflammation markers (ESR= 55 mm/h, CRP=13.7mg/L). Because of the clinical suspicion of GCA, an ultrasound (US) of temporal, facial and axillary arteries followed. Although facial and axillary arteries were normal, vessel wall inflammation was detected in segments of both temporal arteries (**Figure 1**): hypoechoic areas in the vessel wall were displayed in both superficial common arteries and their main branches, forming “halo sign” and resulting in disturbance of blood flow. Based on these findings, a biopsy of the left temporal artery (TAB) was ordered and then treatment with oral methylprednisolone 32mg/day started. There was a rapid improvement in clinical symptoms and laboratory parameters. Therefore, gradual taper of steroids was decided.

**Figure 2.**
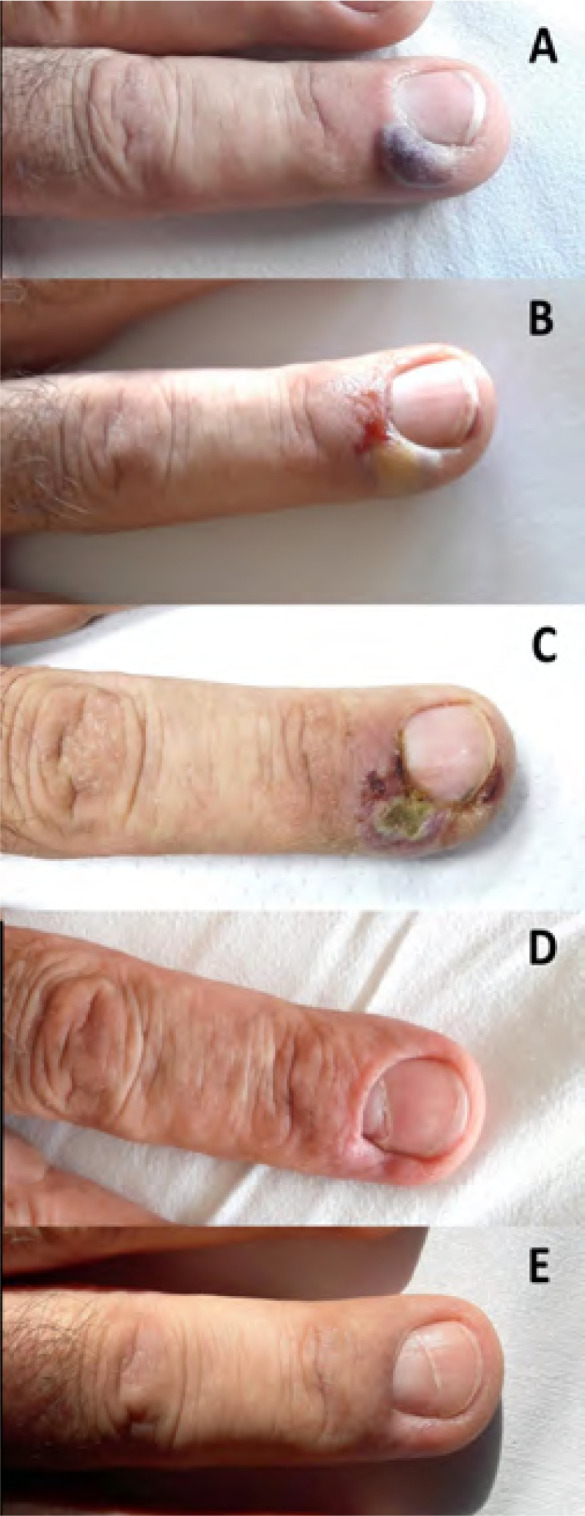
Cutaneous necrosis at distal phalanx of the left middle finger. The progress of the lesion is shown three days before the 1st intravenous cyclophosphamide (A), the day of the infusion (B), four days after the infusion (C), at the second infusion (one month later - D) and at the third infusion (two months later - E).

One month later, while the patient was receiving methylprednisolone 24mg/day, he complained of malaise, dry cough, and recent-onset weakness of his left foot. He could recall having a mild dry cough during the last months, but at this time point the cough exacerbated. From clinical examination he had bilateral (more pronounced in the left side) crackles in chest auscultation and “drop foot” in his left leg. Skin necrosis was developed in some fingertips of his hands the next days (**Figure 2A-C**). Chest computed tomography revealed ground-glass infiltrates, bilaterally and inflammatory markers rose again. To be noted, renal function was normal (serum creatinine=0.8 mg/dL, no red blood cells, casts or proteinuria in urinalysis). From an immunological screening, perinuclear ANCA (p-ANCA) and anti-myeloperoxidase antibody (anti-MPO) were positive (56.7 U/mL, normal values <1U/mL). Meanwhile, TAB turned to be “without significant abnormal findings”. Taking into account the clinical picture and the immunological profile, the diagnosis of MPA was made. Three monthly pulses of intravenous cyclophosphamide 1g (0.5g/m^2^) were administered, accompanied by 3 intravenous pulses of 1000mg methylprednisolone, followed by switch to oral methylprednisolone 32mg/day. Trimethoprim/sulfamethoxazole prophylaxis for *P. jirovecii* (800/160mg 3 times per week) and alendronate 70mg/week with daily cholecalciferol and calcium supplementation for secondary osteoporosis prevention were also given. Induction treatment led to clinical (**Figure 2D-E**), imaging and laboratory improvement and maintenance therapy with daily azathioprine 2mg/kg and gradual tapering of steroids followed. Two years later, the patient is still in remission under azathioprine monotherapy; lung infiltrates have resolved, skin lesions are healed and right foot functionality returned to normal.

## DISCUSSION

Prompt diagnosis of GCA is essential, as delay of treatment can lead to irreversible complications, such as blindness, stroke, and scalp necrosis. According to current EULAR guidelines for imaging in large-vessel vasculitides, temporal and axillary US are the first-line imaging modality for suspected GCA.^2^ In this line, some experts have introduced the “fast-track US clinic” in everyday clinical practice^3^; patients with clinical suspicion of GCA undergo immediate imaging examination (usually US), and if it turns to be positive for vessel inflammation, treatment is initiated as soon as possible. In our case, the patient had vessel wall inflammation in temporal arteries, as shown in US. It has been previously reported that false positive results in temporal US can be found in AAV.^4^ This is more pronounced in patients that “halo sign” is detected in only one branch of the temporal arteries.^4^

To be noted, TAB in our patient did not have significant findings. Despite its high specificity, TAB exhibits a moderate sensitivity, lower than US, in detecting inflammation of temporal artery wall.^5^ This can be attributed mainly to the non-continuous insult of the vessel wall by GCA and to inadequate specimen length acquired by the surgeon. Moreover, it seems that US guidance improves the sensitivity of TAB.^6^ In our case, region of which the sample was obtained was *a priori* indicated with US. TAB was of adequate length (1.1cm). Nevertheless, TAB was not indicative of vasculitis. This implies that either the included vessel areas were not inflamed or that minor non-specific inflammatory lesions were not identified. Thus, special attention should be paid by pathologists to recognize minor infiltrates in the wall of the temporal artery that can possibly indicate diagnoses other than GCA.

Except from GCA, other vasculitides can sometimes cause inflammation in temporal arteries.^7–15^ A recently published case of an elderly man with bitemporal headache, jaw claudication, right-eye vision loss, and severe glomerulonephritis with positive p-ANCA showed that MPA can insult temporal and ophthalmic arteries.^8^ A MPA case of a 65-year-old patient with unilateral temporal headache, scalp tenderness, jaw claudication, mononeuritis and mild glomerulonephritis has seen the light some years ago.^11^ Tanaka et al. reported a case of a 81-year-old man with bilateral temporal headache, fever, interstitial pneumonia and glomerulonephritis, with a positive TAB for vasculitis, that turned out to be MPA.^12^ Suyama et al. reported a case of a patient with headache, fever and PMR that had positive anti-MPO antibodies and was treated as MPA.^7^ TAB in that patient revealed inflammation of vasa vasorum of the temporal artery. Although periadventitial small-vessel vasculitis (SVV) or isolated vasa vasorum vasculitis in TAB have been considered as part of the histopathologic spectrum of GCA,^16^ some of these patients might have AAV or other systemic vasculitides.^9^ Importantly, in a retrospective analysis of 120 cases of histological temporal arteritis and systemic necrotizing vasculitis, 2.5% had MPA.^10^ **Table 1** collectively illustrates the published case reports with temporal arteritis in the context of MPA.

**Table 1.** Case reports of temporal vasculitis in MPA patients and their main clinical manifestations.

**Case No**	**Cranial Manifestations**	**Extra-cranial Manifestations**	**Reference**
1	headache, jaw claudication	fever, PMR, ILD, PNS, skin	Present case
2	headache, scalp tenderness	fever, PMR	(7)
3	headache, jaw claudication, visual disturbance	fever, ILD, GN	(8)
4	headache, jaw claudication, scalp tenderness	ILD, GN, myositis	(11)
5	Headache	fever, ILD, GN	(12)
6	headache, jaw claudication	fever, ILD, GN, PNS, skin, gastroepiploic artery rupture	(13)
7	headache, jaw claudication, visual disturbance	GN, PNS, skin	(14)
8	Headache	GN, PNS	(15)

No: number, PMR: polymyalgia rheumatic, ILD: interstitial lung disease, GN: glomerulonephritis, PNS: peripheral nervous system involvement

The patient presented here had also PMR symptoms. In one retrospective study, 13% of patients with systemic SVV initially presented as PMR and had more mild renal involvement than the rest.^17^ Notably, kidneys were not affected in our patient. A meticulous clinical examination and laboratory testing might help physicians to distinguish systemic SVV from “isolated” PMR. Interestingly, in our case corticosteroids did not inhibit the development of MPA, but only delayed its full presentation. This can be explained by the fact that glucocorticoid monotherapy is generally not recommended for induction therapy of MPA because of lower remission and higher relapse rates.^18^

In conclusion, this case underlines that AAV can affect the cranial arteries, presenting with symptoms that can mimic GCA. Close monitoring of patients with “cranial” symptoms is needed; relapse or lack of response to treatment might question the initial diagnosis of GCA. A high clinical suspicion from clinicians and pathologists is necessary to early recognize this condition and initiate suitable treatment to prevent serious complications for the patients.
